# Surgical Notes

**Published:** 1858-07

**Authors:** W. W. Jones

**Affiliations:** Toledo, Ohio


### ART. V.— *Surgical Notes. Dislocations of the Hip.* By W. W. Jones, M. D., Toledo, Ohio.

*Sept.* 2d, 1853. Michael Glancy, an Irish laborer, aged 35, while at work at the railroad excavation near this city, was struck by falling earth; found him two hours after the accident at home, where he had been carried upon a litter, suffering intensely at the right hip; leg shortened 2½ inches and foot turned inwards; great trochanter upon the dorsum ilii. Tried reduction by Dr. Reid's method, which I had read a short time before, but could not succeed in reducing the bone. Having no pullies nor adjuster at hand, I procured a rope and applied what I had often seen boatmen use to warp boats, which they term a Spanish windlass, consisting of an upright and a horizontal pole interlocked in the rope; two men walking around with the horizontal pole, wind the rope on the upright one, which is held up by another. With this kind of a windlass any amount of extension can be regularly and steadily applied, the only materials necessary being a rope and a wood pile or a stiff walking stick. Having applied the apparatus and made the patient fast, two men easily pulled it in place while I manipulated the leg. Patient was very sore and lame for a week or more, and could not work for a month afterwards.

*May* 25th, 1854. James McCafferty, laborer, aged 25, strong and muscular, in attempting to jump on the cars while in motion, missed his footing and fell or was drawn under the wheels. Found him with his hand cut and bruised in several places, clavicle fracturad, and left hip dislocated, leg shortened 2½ inches. With the assistance of Drs. Daniels and Bostwick, of this city, and Woodward, of Fort Wayne, attempted reduction by Reid's method several times, without being able to accomplish it. Applied Jarvis' adjuster, and succeeded in the reduction after using great force, so much as to bend the racket bar of the Instrument. Patient very restless, and pulse very rapid, which continued until he died, three hours after, and about four hours after the injury, from the shock. No post mortem could be obtained.

*June* 19th, 1854. Laclin Hunter, a sailor and Scotchman, aged 37, had been at work in the hold of a vessel, amongst some timber, that had fallen against him; found him in the cabin of the vessel, on the floor, with right knee and foot turned inwards and shortened, and the great trochanter upon the dorsum ilii, lay stiff. Diagnosed it as dislocation of the right hip, upwards and back. Attempted reduction by Dr. Reid's method of flexion; carried the knee flexed as near the abdomen as possible, and as the knee passed to the right side of the body, the head of the bone went off with a snap, as I supposed, into the acetubulum, but no, I could not straighten the limb! the head of the bone was not in the acetubulum, but in the *obturator foramen;* the knee stood outwards and upwards and immovable; the patient tortured and myself in a quandary; undertook to pull it out, in the direction of the bone, with the assistance of three men, who were present; could make no impression upon it; thought of the wedge, recommended, I think, by Dr. Brainard, of Chicago, in a similar dislocation, but it seemed to me impracticable, as there was no place to apply it. It occurred to me that I could reduce it by the same means that had caused the difficulty. I tried it, and turned the knee around back, precisely where it was before, with very little difficulty, describing the same curve, and found I had the dislocation back upon the dorsum ilii again. The next time I carried the knee up to an angle of forty-five degrees from the abdomen, then abducted, and this time the bone slipped into the acetabulum, and I had the satisfaction of seeing my patient all right.

*Sept.* 15th, 1854. Patrick Kearney, laborer, aged 38, injured at the railroad excavation, near the city; found him at home, where he had been carried, right hip dislocated on the dorsum ilii; leg shortened 2 inches; stiff and turned in; flexed the leg upon the thigh and carried the knee over the left pelvis up to an angle of about 50 degrees with the abdomen, then abducted, and the bone slipped into its place in the acetubulum. Patient was about in a few days.

I have notes of two other cases, reduced in the same manner as the last' with no other variation than date, age, &c.

